# Supplementary material for: Hypoxic microenvironment shapes HIV-1 replication and latency
Source: Commun Biol. 2020 Jul 14;3:376. doi: 10.1038/s42003-020-1103-1 (PMC7360605; doi:10.1038/s42003-020-1103-1)
Supplement: Supplementary file 1 — Supplementary Information [file 42003_2020_1103_MOESM1_ESM.pdf]

SUPPLEMENTAL INFORMATION

Hypoxic microenvironment shapes HIV-1 replication and latency.

Xiaodong Zhuang, Isabela Pedroza-Pacheco, Isabel Nawroth, Anna E Kliszczak,
Andrea Magri, Wayne Paes, Claudia Orbegozo Rubio, Hongbing Yang, Margaret Ashcroft,
David Mole, Peter Balfe, Persephone Borrow and Jane A McKeating.

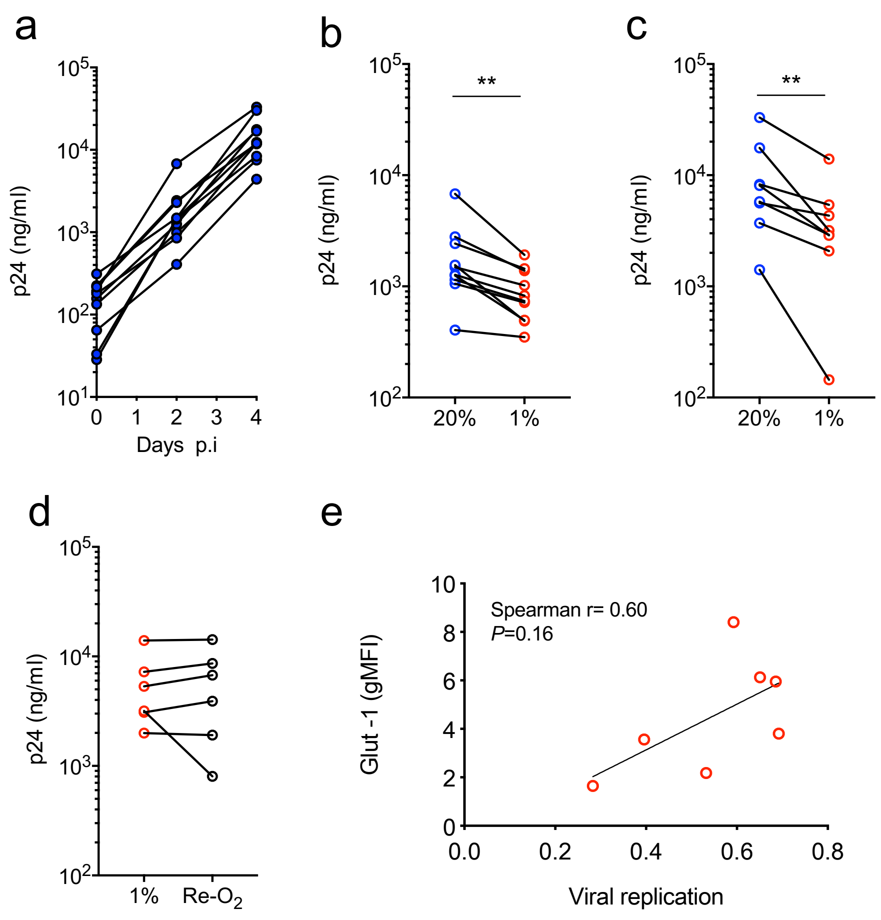

Supplementary Figure 1. Replication of HIV-1 NL4.3-Bal in CD8-depleted activated PBMCs. Activated CD8-depleted PBMC were infected with HIV-1 NL4.3-Bal and cultured under 20% or 1% O_2 conditions and viral replication was assessed at 0, 2 and 4 days post-infection by measuring supernatant p24 antigen levels. In some experiments, cells under 1% O_2 were re-oxygenated for the last 2 days of culture (Re-O_2). **(a)** Viral replication over time in cells from different donors cultured in 20% O_2. The results shown at each timepoint are the average supernatant p24 values from two replicate wells. Each line represents data from an individual donor (n=10). **(b, c)** Supernatant p24 antigen levels in cells from each donor cultured under 20% or 1% O_2 conditions after **(b)** 2 days of infection, or **(c)** 4 days of infection (n=7-10). **(d)** Supernatant p24 antigen levels in cells from each donor cultured for 4 days under 1% O_2 or re-oxygenated for the last 2 days of culture (n=6). Each symbol represents data from an individual donor where two independent biological replicates were analysed. Paired samples are indicated (n=6-10, Wilcoxon matched-pairs signed rank test). **(e)** Spearman Correlation of levels of Glut-1 expression and HIV-1 replication (as extracellular p24 antigen levels) under normoxic conditions relative to values in 20% O_2 after 2 days of culture. Each symbol represents data from an individual donor.

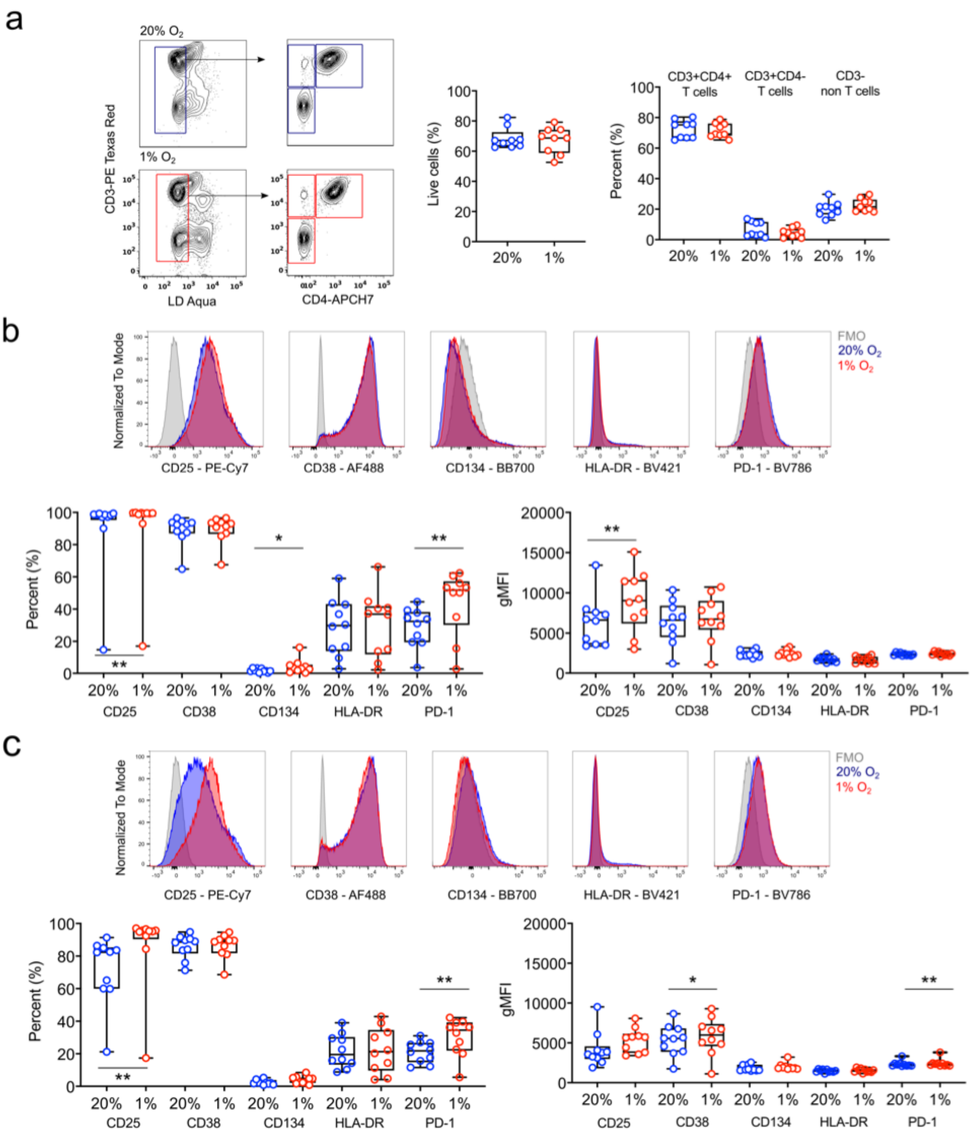

Supplementary Figure 2. CD4 T cell viability and activation are not impaired under low oxygen tension. Activated CD8 depleted PBMC were incubated (without infection) under 20% or 1% O_2 for 2 or 4 days. **(a)** Cell viability was assessed on day 4 by flow cytometry using a live-dead stain, and the proportion of T cells within the live cell population was evaluated. Histogram plots illustrating staining of cells from one representative donor are shown on the left, and summary plots of the percentage of live cells and proportion of CD3+CD4+ and CD3+CD4- T cells and CD3- non-T cells within all live cells are shown on the right (n=10, mean + SEM, Wilcoxon matched-pairs signed rank test). **(b, c)** T cell activation was assessed by evaluating CD25, CD38, CD134, HLA-DR and PD-1 expression on days 2 **(b)** and 4 **(c).** Histogram plots illustrating staining of cells from one representative donor at day 2 and day 4 are shown, and together with summarised data showing the percentage of cells expressing each marker within the live CD3+CD4+ population and level of intensity of staining (geometric mean fluorescence intensity (gMFI)) of this population at 2 and 4 days post-activation (n=10, mean + SEM, Wilcoxon matched-pairs signed rank test). Each symbol represents data from one individual donor.

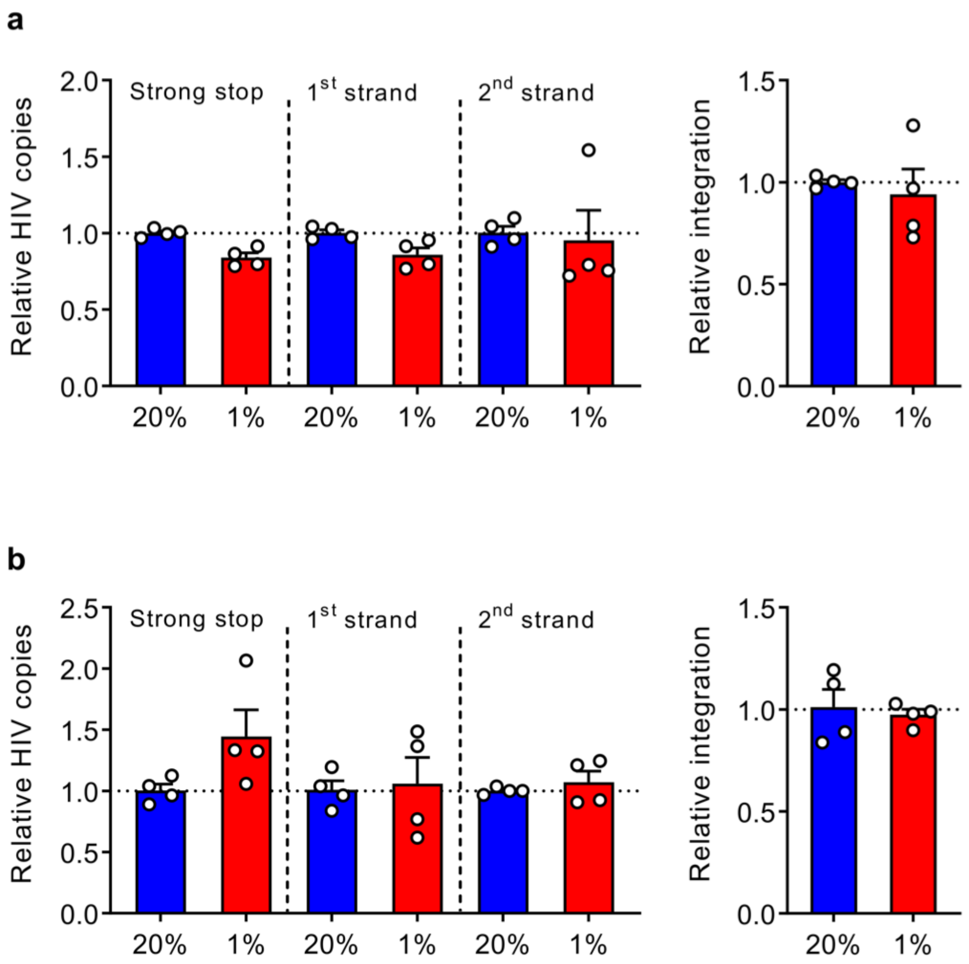

Supplementary Figure 3. Low oxygen conditions have no effect on the early steps of viral life cycle and HIV integration events in 1G5 and TZM-bl cells. (a) 1G5 or **(b)** TZM-bl cells were infected with HIV NL4.3-Bal under 20% or 1% O_2 for 48h. HIV strong stop, 1st and 2nd strand products along with the number of HIV integrated copies in the hypoxic cultures are expressed relative to the normoxic controls. Each symbol represents data from one biological replicate (n=4; mean ± SEM, Wilcoxon matched-pairs signed rank test).

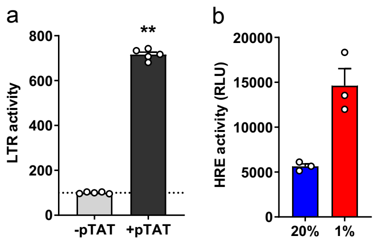

Supplementary Figure 4. Expression of pTAT in TZM-bl cells increases HIV-LTR activity; Low oxygen increases HRE promoter activity. (a) TZM-bl cells were transfected with a control plasmid or pTAT and LTR activity was assessed 24h later by evaluating luciferase expression in cell lysates and the data expressed relative to the normoxic controls (n = 5, mean ± SEM, Mann-Whitney analysis). **(b)** HRE-Luc reporter were transfected into Jurkat cells and cultured in 20% or 1% O2 conditions for 48h. Luciferase values (relative light units, RLU) are reported (n = 3, mean ± SEM).

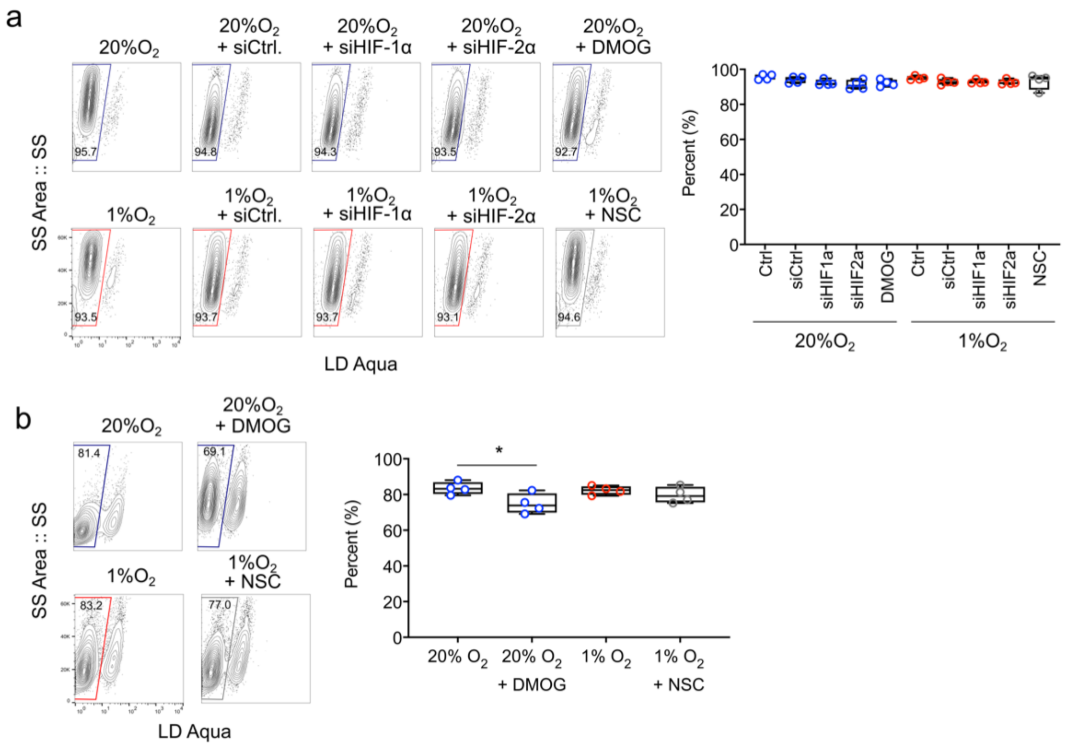

Supplementary Figure 5. Evaluation of the effect of HIF silencing and NSC or DMOG treatment on cell viability. (a) Jurkat cells were cultured at 20% or 1% O_2 with HIF-1α or HIF-2α silencing or treatment with DMOG or NSC. Cell viability was assessed after 24 hours by flow cytometry using a live-dead stain. Representative plots are shown on the left, and summarised data from cells from 4 different experiments is shown on the right. (n=4, mean ± SEM, Kruskal-Wallis with Dunn's MCA analysis). **(b)** Activated primary CD4 T cells were cultured at 20% or 1% O_2 and treated with DMOG or NSC and cell viability was assessed after 24 hours by flow cytometry using a live-dead stain. Representative plots are shown on the left, and summarised data from cells from different donors is shown on the right (n=4; mean ± SEM, Wilcoxon matched-pairs signed rank test).

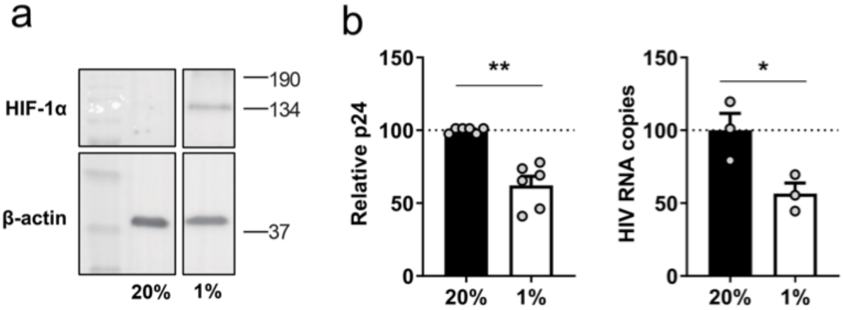

Supplementary Figure 6. Effect of low oxygen on TNFα reactivation of HIV-1 in ACH2 cells. **(a)** ACH2 cells cultured under 20% or 1% O_2 conditions were assessed for HIF-1α expression by western blotting. **(b)** ACH2 cells were activated with TNFα under 20% or 1% O_2 conditions and viral replication assessed 48h later by measuring extracellular p24 antigen or quantitative real-time PCR of HIV RNA and the data expressed relative to the normoxic controls (n = 3-6, mean + SEM, Unpaired t test).

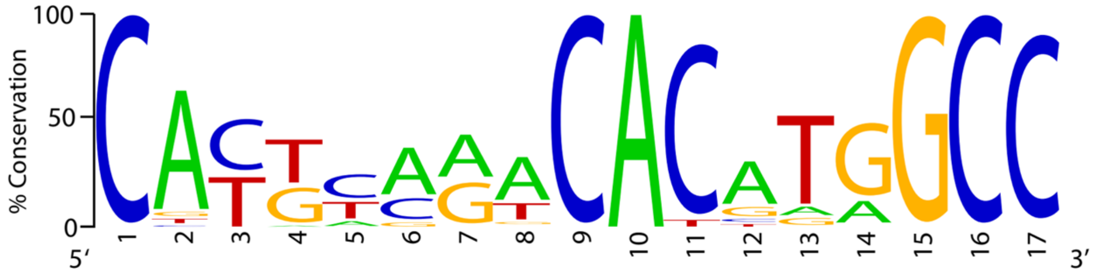

Supplementary Figure 7. HRE conservation in HIV-1 strains found in early infection. A search of the LANL database (April 2019) identified 84 sequences derived from patients sequenced in acute/early infection, as defined by a Fiebig score of 1-5, where LTR sequences were available. The sequences were derived from HIV-1 subtypes A (27), B (28), C (20) or D (5), with the remaining 5 sequences representing recombinant virus types. The image shows the same LTR region as depicted in Fig.5a and highlights that 43 of 84 sequences (51%) contained a conserved HRE.

Supplementary Table 1. Primers used in study. Oligonucleotide sequences are listed below. Oligos used for mutagenesis are shown in lower case with the altered bases shown in bold.

	Forward 5' – 3'	Reverse 5' – 3'
HIV strong stop	oHC64 (TAACTAGGGAACCCACTGC)	oHC65 (GCTAGAGATTTTCCACACTG)
	Taqman Probe oHC66 (FAM-ACACAACAGACGGGCACACACTA-BHQ1)	
HIV 1st strand	HIV-fst-f1 (GAGCCCTCA GATCCTGCATAT)	oHC65 (GCTAGAGATTTTCCACACTG)
	Taqman Probe oHC66 (FAM-ACACAACAGACGGGCACACACTA-BHQ1)	
HIV 2nd strand	oHC64 (TAACTAGGGAACCCACTGC)	gag-m661-as (CTGCGTCGAGAGAGCTCCTCTGGTT)
	Taqman Probe oHC66 (FAM-ACACAACAGACGGGCACACACTA-BHQ1)	
HIV integration	Alu-F (GCCTCCCAAAGTGCTGGGATTACAG)	gag-R (GTTCCTGCTATGTCACTTCC)
	LTR-F(TTAAGCCTCAATAAAGCTTGCC)	LTR-R(GTTCGGGCGCCACTGCTAGA)
	Taqman Probe oHC66 (FAM-ACACAACAGACGGGCACACACTA-BHQ1)	
HIV RNA	PATH-HIV1 KIT, PRIMER DESIGN LTD. UK	
HRE deletion	atgcagctctcgggcatgaaatgctaggcg	cgcctagcatttcatgcccgagagctgcat
HRE mutant 1	atgcagctctcgggc**catatg**atgaaatgctaggcg	cgcctagcatttcatcatatggcccgagagctgcat
HRE mutant 2	ccggatgcagctctcgggct**cacgg**atgaaatgctaggcggctg	cagccgcctagcatttcatccgtgagcccgagagctgcatccgg
HIF1β ChIP LTR-HRE	TCGATGTCAGCAGTTCTTGAAGTA	CCTGAGAGAGAAGTGTTAGAGTGG
HIF1β ChIP LTR non-HRE	GTAGTTCTGCCAATCAGGGAAGTA	GACTTACAAGGCAGCTGTAGATCT
HIF1β ChIP CA9	TCTCGTTTCCAATGCACGTACAGC	AGTGACAGCAGCAGTTGCACAGT
HIF1β ChIP VEGFA	GGGAGCCCCTAGGCCACTA	AGGCCGTGGACCCTGGTA
HIF1β ChIP β-actin	AGATGTGGATTAGCAAGCAGG	GCTTATTCCAGTTTCGTGAGGC
