# Supplementary material for: Hypoxic microenvironment shapes HIV-1 replication and latency
Source: Commun Biol. 2020 Jul 14;3:376. doi: 10.1038/s42003-020-1103-1 (PMC7360605; doi:10.1038/s42003-020-1103-1)
Supplement: Supplementary file 2 — Description of additional supplementary items [file 42003_2020_1103_MOESM2_ESM.pdf]

Description of additional supplementary files

Supplementary Data 1: Source data
