# Supplementary material for: Hypoxic microenvironment shapes HIV-1 replication and latency
Source: Commun Biol. 2020 Jul 14;3:376. doi: 10.1038/s42003-020-1103-1 (PMC7360605; doi:10.1038/s42003-020-1103-1)
Supplement: Supplementary file 4 — Reporting Summary [file 42003_2020_1103_MOESM4_ESM.pdf]

nature research

Corresponding author(s): Jane A McKeating

Last updated by author(s): May 28, 2020

Reporting Summary

Nature Research wishes to improve the reproducibility of the work that we publish. This form provides structure for consistency and transparency in reporting. For further information on Nature Research policies, see Authors & Referees and the Editorial Policy Checklist.

Statistics

For all statistical analyses, confirm that the following items are present in the figure legend, table legend, main text, or Methods section.

n/a	Confirmed	
☐	☒	The exact sample size (*n*) for each experimental group/condition, given as a discrete number and unit of measurement
☐	☒	A statement on whether measurements were taken from distinct samples or whether the same sample was measured repeatedly
☐	☒	The statistical test(s) used AND whether they are one- or two-sided<br>*Only common tests should be described solely by name; describe more complex techniques in the Methods section.*
☒	☐	A description of all covariates tested
☒	☐	A description of any assumptions or corrections, such as tests of normality and adjustment for multiple comparisons
☐	☒	A full description of the statistical parameters including central tendency (e.g. means) or other basic estimates (e.g. regression coefficient) AND variation (e.g. standard deviation) or associated estimates of uncertainty (e.g. confidence intervals)
☐	☒	For null hypothesis testing, the test statistic (e.g. *F*, *t*, *r*) with confidence intervals, effect sizes, degrees of freedom and *P* value noted<br>*Give P values as exact values whenever suitable.*
☒	☐	For Bayesian analysis, information on the choice of priors and Markov chain Monte Carlo settings
☒	☐	For hierarchical and complex designs, identification of the appropriate level for tests and full reporting of outcomes
☒	☐	Estimates of effect sizes (e.g. Cohen's *d*, Pearson's *r*), indicating how they were calculated

Our web collection on statistics for biologists contains articles on many of the points above.

Software and code

Policy information about availability of computer code

Data collection	*Provide a description of all commercial, open source and custom code used to collect the data in this study, specifying the version used OR state that no software was used.*
Data analysis	All statistical analyses were performed using GraphPad Prism 7 and 8.

For manuscripts utilizing custom algorithms or software that are central to the research but not yet described in published literature, software must be made available to editors/reviewers. We strongly encourage code deposition in a community repository (e.g. GitHub). See the Nature Research guidelines for submitting code & software for further information.

Data

Policy information about availability of data

All manuscripts must include a data availability statement. This statement should provide the following information, where applicable:

- Accession codes, unique identifiers, or web links for publicly available datasets
- A list of figures that have associated raw data
- A description of any restrictions on data availability

The authors declare that all data supporting the findings of this study are available within the article and its Supplementary Information files, or are available from the authors upon request.

Field-specific reporting

Please select the one below that is the best fit for your research. If you are not sure, read the appropriate sections before making your selection.

☒ Life sciences ☐ Behavioural & social sciences ☐ Ecological, evolutionary & environmental sciences

For a reference copy of the document with all sections, see nature.com/documents/nr-reporting-summary-flat.pdf

Life sciences study design

All studies must disclose on these points even when the disclosure is negative.

Sample size	Sample size was chosen consistent with the previous literature utilising similar assays. For most experiments this was achieved using triplicate measurements. Where used, higher replicate numbers are indicated in the text.
Data exclusions	No data was excluded
Replication	Biological replicates were performed as described in the main text.
Randomization	No randomization was performed
Blinding	Blinding was not relevant to this study

Reporting for specific materials, systems and methods

We require information from authors about some types of materials, experimental systems and methods used in many studies. Here, indicate whether each material, system or method listed is relevant to your study. If you are not sure if a list item applies to your research, read the appropriate section before selecting a response.

Materials & experimental systems

n/a	Involved in the study	
☐	☒	Antibodies
☐	☒	Eukaryotic cell lines
☒	☐	Palaeontology
☒	☐	Animals and other organisms
☒	☐	Human research participants
☒	☐	Clinical data

Methods

n/a	Involved in the study	
☒	☐	ChIP-seq
☐	☒	Flow cytometry
☒	☐	MRI-based neuroimaging

Antibodies

Antibodies used	Antibodies specific for HIF-1α were purchased from BD Biosciences and anti-HIF-2α antibody provided by the Ratcliffe laboratory (University of Oxford). Cells were stimulated with soluble human anti-CD3 (R&D; clone UCHT1) and 0.1 ug/ml soluble human anti-CD28 (Life Technologies; clone CD28.2) and stained with the following antibodies: anti-CD4 APC-H7 (SK3, BD Biosciences); anti-CD38 Pecy5 (HIT2, Biolegend); anti-HLA-DR BV421 (L243, Biolegend); anti-CD4 APC-H7 (SK3, BD Biosciences); anti-CD38 Pecy5 (HIT2, Biolegend), anti-HLA-DR BV421 (L243, Biolegend), anti-CD3 PE-Texas Red (S4.1, Life Technologies) and anti-GLUT-1 APC (202915, R&D).<br><br>In addition cells were stained with an antibody cocktail: CCR5 Pe-Dazzle 594 (J418F1, BioLegend), CCR6 PE (11A9, BD Biosciences) , CCR7 BV650 (G043H7, BioLegend), CD134 BB700 (ACT35, BD Biosciences), CD25 PE-Cy7 (2A3, BD Biosciences), CD38 AF488 (HIT2, BioLegend), CD4 APC-H7(SK3, BD Biosciences), CD45RA BV711 (HI100, BioLegend), CXCR3 PE-Cy5 (1C6/CXCR3, BD Biosciences), CXCR4 BV605 (12G5, BioLegend), HLA-DR BV421 (L243, BD Biosciences), PD-1 BV786 (EH12.1, BD Biosciences), CD3 APC-R700 (UCHT1, BD Biosciences) and Foxp3 APC (PCH101, Life Technologies).
Validation	All commercially available antibodies were validated by their respective manufacturers.

Eukaryotic cell lines

Policy information about cell lines

Cell line source(s)	1G5 cells were provided by Professor Ariberto Fassati (University College London, London, UK); TZM-bl and ACH2 cells were provided by Professor Bill Paxton (University of Liverpool, Liverpool, UK); Jurkat cells and J-Lat clone 6.3 were provided by Professor Xiaoning Xu (Imperial College, London) and 293T cells were obtained from ATCC.

Authentication	None of the cell lines were authenticated
Mycoplasma contamination	Mycoplasma testing was performed on cell lines and verified as negative.
Commonly misidentified lines (See ICLAC register)	None

Flow Cytometry

Plots

Confirm that:

☒ The axis labels state the marker and fluorochrome used (e.g. CD4-FITC).

☒ The axis scales are clearly visible. Include numbers along axes only for bottom left plot of group (a 'group' is an analysis of identical markers).

☒ All plots are contour plots with outliers or pseudocolor plots.

☒ A numerical value for number of cells or percentage (with statistics) is provided.

Methodology

Sample preparation	Peripheral blood mononuclear cells (PBMCs) were isolated from leukapheresis samples from healthy donors (NHS Blood and Transplant Service). PBMCs were depleted of CD8+ cells using CD8 microbeads (Miltenyi Biotec).
Instrument	Cell fluorescence was detected on a BD Fortessa X20 flow cytometer using BD FACSDiva8.0 (BD Bioscience)
Software	Data analyzed using FlowJo 10 (TreeStar).
Cell population abundance	Cell viability was assessed by flow cytometry using a live-dead stain. Contour plots with outliers illustrating staining of cells from one representative donor are shown in Supplementary figure 2 and summary plots of the percentage of live cells and proportion of live cells within CD3+CD4+ and CD3+CD4- T cells and CD3- non-T cells presented.
Gating strategy	Contour plots with outliers illustrating staining of cells from one representative donor are shown in Supplementary figure 2 and a summary of the percent of expressing cells (chemokine and activation markers) within the live CD3+CD4+ population and level of intensity of staining (gMFI) of this population after 2 (n=10) and 4 (n=10) days post-activation is shown (mean + SEM, Wilcoxon matched-pairs signed rank test).

☒ Tick this box to confirm that a figure exemplifying the gating strategy is provided in the Supplementary Information.
